# Screening for assessment of diabetic peripheral neuropathy in Diamantina, Minas Gerais-Brazil

**DOI:** 10.1186/1758-5996-7-S1-A24

**Published:** 2015-11-11

**Authors:** Edson da Silva, Alline Sancha Nascimento Fernandes, Sabrina Campos Ferraz, Rafael Leite Alves, Flávio Mariz Freitas, Luciana Duarte Novais da Silva, Clynton Lourenço Corrêa

**Affiliations:** 1 Universidade Federal dos Vales do Jequitinhonha E Mucuri – UFVJM, Diamantina, Brazil

## Background

Neuropathy is a common complication of type 1 and type 2 diabetes mellitus (DM) that contributes to both mortality and morbidity among the diabetic population leading to substantial physical, physiological and financial burden for the patients and community at large. Diabetic foot complications are a global problem with increasing incidence of DM worldwide.

## Objectives

The aim of the study was to evaluate the sensitivity level of the feet, lower limb strength and balance (static and dynamic) in patients with diabetes regarding the presence or not of peripheral neuropathy.

## Materials and methods

This study was carried out in the Diamantina, Minas Gerais-Brazil. This is a prospective study conducted in 27 volunteers, 5 diagnosed as type 1 diabetes (DM1), 10 as type 2 diabetes (DM2). All volunteers underwent a protocol with detailed clinical examination, glycemic index, muscle strength, balance and dynamic mobility, and sensitivity of the feet using the Michigan Neuropathy Screening Instrument and Semmes-Weinstein Monofilaments. After collecting data, Results of both groups were compared with their controls.

## Results

DM1 and DM2 groups had higher levels of blood glucose compared with the respective control (p<0.01; p<0.05, respectively). DM1 group had weight and body-mass index (BMI) lower than DM1C group (p<0.01). The sensitivity studied showed a distribution with predominance of purple monofilament in DM1 and DM1C groups and purple and red monofilaments in DM2 e DM2C groups in different points of the feet. All groups showed changes in muscle strength. However, the groups of subjects with diabetes had a slight deficit of strength in relation to the controls. Only DM2 and DM2C groups presented score indicative of peripheral neuropathy in Michigan Neuropathy Screening Instrument.

## Conclusion

In the present study both DM1 and DM2 groups showed changes in tests and were more evident in DM2 group. However, this finding is not sufficient to indicate the presence of peripheral neuropathy in this population. The duration of diabetes is a factor that suggests severity of symptomatic subjects, for this reason it is interesting to assess and correlate it with other variables in future studies.

